# Homozygous Delta-Beta Thalassaemia With Alpha Thalassaemia and Erythrocytosis- a Rare Case Report

**DOI:** 10.3389/bjbs.2024.13663

**Published:** 2024-11-21

**Authors:** Hala Shokr, Mandeep Kaur Marwah, Hisam Siddiqi, Christine Wright, Sukhjinder Marwah

**Affiliations:** ^1^ Pharmacy Division, School of Health Sciences, Faculty of Biology, Medicine and Health, The University of Manchester, Manchester, United Kingdom; ^2^ Aston Medical School, College of Health and Life Sciences, Aston University, Birmingham, United Kingdom; ^3^ Department of Hematology, Sandwell and West, Birmingham Hospitals National Health Service Trust, West Bromwich, United Kingdom

**Keywords:** delta-beta thalassemia, foetal haemoglobin, RBC indices, thalassemia, inherited blood disorders

## Abstract

In this report, we describe a case of homozygous delta-beta (δβ) thalassaemia, a rare genetic disorder characterized by severe deficiency in delta (δ) and beta (β)-globin chain production, leading to ineffective erythropoiesis and chronic haemolytic anaemia. The patient, a 26-year-old female with δβ-thalassaemia, experienced a miscarriage. High-performance liquid chromatography revealed 89.5% foetal haemoglobin (HbF) and 14.4% glycated HbF. Sebia capillary electrophoresis showed haemoglobin peak of 97.2% and 2.8%. Kleihauer Bekte test indicated a pancellular pattern of foetal cells, while morphology analysis demonstrated microcytic, hypochromic red cells and target cells. Gene analysis confirmed compound heterozygosity for two large deletions in the β-globin gene cluster.

## Introduction

Homozygous δβ-thalassaemia is a rare yet clinically significant haemoglobinopathy, drawing attention for its unique genetic and physiological manifestations [1]. It is characterized by the complete absence or severe reduction of δ and βglobin chain production, an essential component of haemoglobin (Hb), the oxygen-carrying protein in red blood cells (RBCs) [2]. This deficit leads to ineffective erythropoiesis—the impaired production and maturation of RBCs—culminating in chronic haemolytic anaemia [3]. The genetic origin of this condition lies in mutations of both the delta (δ) and β-globin genes located on chromosome 11, which disrupt the normal production of adult haemoglobin (HbA) [4]. Consequently, this abnormality triggers the gamma genes on the affected chromosomes to compensate by increasing the synthesis of HbF, which is normally produced during foetal development but largely replaced by HbA after birth [5, 6].

Clinically, homozygous δβ-thalassaemia and hereditary persistence of foetal haemoglobin (HPFH) often present with features similar to thalassaemia intermedia, typically characterized by mild anaemia [7]. From a diagnostic perspective, high-performance liquid chromatography (HPLC) and capillary electrophoresis—two commonly used diagnostic tools—typically show a predominance of HbF in affected individuals [8]. However, due to the rarity of this condition and its relatively mild clinical presentation, it is frequently underdiagnosed or misidentified.

Globally, thalassaemia syndromes are more prevalent in regions where malaria was or remains endemic, including parts of Africa, the Mediterranean, the Middle East, and Southeast Asia [9]. However, homozygous δβ-thalassaemia is a much rarer condition, with its prevalence varying significantly across different populations [10]. In fact, the exact epidemiology of homozygous δβ-thalassaemia is not well-established, as it is less commonly encountered and studied compared to more prevalent forms like β-thalassaemia or alpha (α)-thalassaemia [11].

Despite its scarcity, homozygous δβ-thalassaemia remains an important condition for clinicians and researchers to understand due to its unique genetic and haematological features, as well as the impact it can have on affected individuals [1, 12]. Its mild presentation can mask the underlying genetic complexity, making accurate diagnosis crucial for appropriate patient management and family genetic counselling.

Most existing research on the impact of thalassaemia syndromes on pregnancy primarily focuses on beta-thalassaemia major and intermedia, with relatively little attention given to pregnancies in women carrying thalassaemia traits. Although there is a growing awareness of the haemoglobin E syndromes, data on pregnancy outcomes for women affected by these conditions remains sparse. The limited information available suggests a heightened risk of perinatal loss and intrauterine growth restriction (IUGR) among these patients [13]. However, when it comes to obstetric management, the primary determinants of maternal and foetal risk, as well as pregnancy outcomes, are largely influenced by factors such as the severity of anaemia, existing maternal complications, and any organ damage resulting from iron overload. The effects of severe anaemia on pregnancy, combined with pre-existing organ damage, further complicate management [14].

In this case report, we present the haematological findings of a young female patient diagnosed with homozygous δβ-thalassaemia. By examining her case, we aim to shed light on the diagnostic nuances of this rare disorder, offering a deeper understanding of its clinical course, diagnostic markers, and the importance of recognizing it in a broader spectrum of haemoglobinopathies.

## Case Description

A 26-year-old black African female referred by her general practitioner (GP) to south-west Birmingham NHS Hospitals Trust, for a full blood count (FBC) and investigation of sickle cell based on the patient’s report of a positive family history. Upon examination the patient was not pregnant, however 2 years ago, she experienced an unexplained miscarriage in the first trimester.

Medical records indicated that the patient had no history of receiving blood transfusions, but iron therapy was administered to manage low mean corpuscular volume (MCV) and mean corpuscular haemoglobin (MCH) levels.

## Discussion and Diagnostic Assessment

In our case, this variant of thalassemia was diagnosed at 26 years of age. Mondal et al. reported a wide age range for affected individuals, from 5 months to 72 years [15]. Typically, these cases exhibit mild to moderate anaemia, with RBCs counts varying from slightly elevated to normal or decreased [16]. In this case the clinical assessment showed no evidence of anaemia or fatigue. To investigate potential underlying causes of the miscarriage, the GP ordered blood tests including a FBC, hematinic profile, urea and electrolyte levels, liver function tests, and a hemoglobinopathy screen (Table 1).

**TABLE 1:** Patient clinical characteristics.

Parameters	Patient value	Reference range
WBCs (10*^9^/L)	6.1	4–11
RBCs (10*^12^/L)	+7.05	3.8–5.2
Hb (g/L)	+170	115–160
HCT (L/L)	0.49	0.37–0.45
MCV (fL)	−69.8	80–100
MCH (pg)	−24.1	27–32
MCHC (g/L)	346	310–350
PLT (10*^9^/L)	269	150–450
B12 (ng/L)	+981	188–883
Folate (ug/L)	4.0	3.1–20
Ferritin (ug/L)	18	10–300
Urea (mmol/L)	3.6	2.5–7.8
Sodium (mmol/L)	140	135–145
Potassium (mmol/L)	5.0	3.5–5.3
Creatinine (µmol/L)	97	45–110
eGFR (mL/min/1.73 m^2^)	65	—
Albumin (g/L)	44	35–50
Bilirubin (µmol/L	12	<21
Alkaline Phosphatase (IU/L)	142	30–130

WBCs, white blood cells; RBC, red blood cells; Hb, haemoglobin; HCT, haematocrit; MCV, mean corpuscular volume; MCH, mean corpuscular haemoglobin; MCHC, mean corpuscular haemoglobin concentration; PLT, platelets; B12, vitamin B12; eGFR, estimated glomerular filtration rate.

Heterozygous δβ-thalassemia is usually characterized by elevated levels of HbF, ranging from 5% to 20%, along with thalassaemic red blood cell indices, such as high RBC count and low MCV and MCH, while HbA_2_ levels are normal or reduced [17]. In contrast, the homozygous form presents with similar thalassaemic red cell indices (high RBC count, low MCV and MCH, and elevated reticulocyte count) but is distinguished by very high HbF levels and reduced HbA_2_. Additionally, it often shows biochemical signs of haemolytic anaemia, such as increased indirect bilirubin and decreased serum haptoglobin. Hepatosplenomegaly may or may not be observed [18].

The patient’s blood analysis revealed a slight elevation in RBCs count, high Hb concentration, reduced MCV and a microcytic, hypochromic appearance, with target cells present, consistent with findings from other studies [17, 19].

The patient had also elevated vitamin B12 levels, with folate and ferritin levels within the normal reference range. Blood urea and electrolyte levels were also normal, but alkaline phosphatase levels were elevated (Table 1).

**FIGURE 1:**
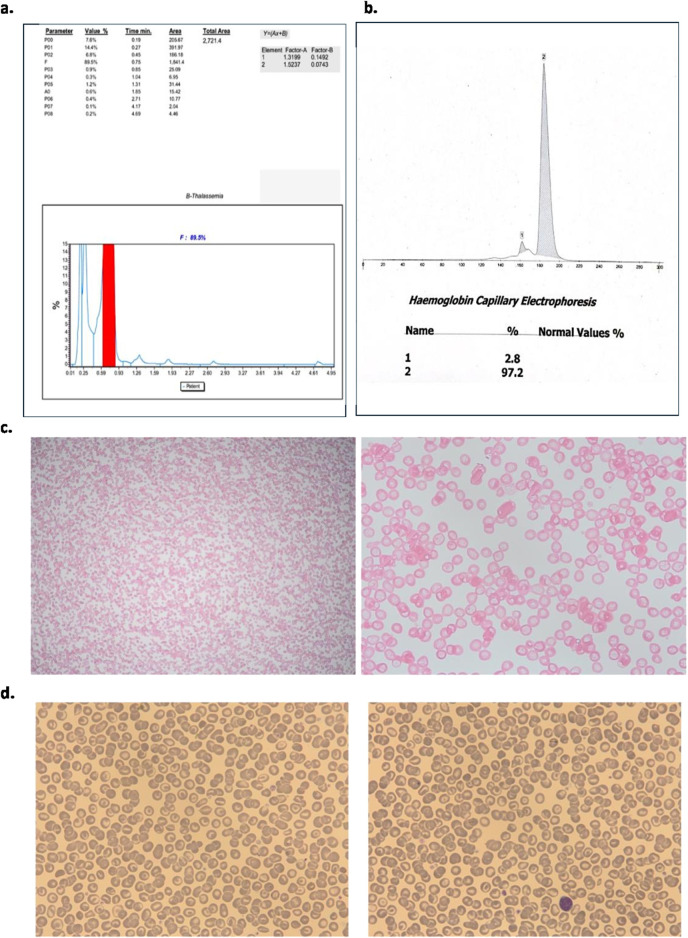
**(A)** Tosoh-G11 Chromatogram demonstrated foetal haemoglobin as a major peak and minor peak for glycated foetal haemoglobin; **(B)** Sebia Capillary electrophoresis; **(C)** Kleihauer Bekte showing pancellular expression; **(D)** Morphological analysis.

The patient’s blood sample was further analysed using two different analytical instruments: HPLC (Tosoh-G11) and Sebia Capillary Electrophoresis. The HBLC analysis detected 89.5% HbF and 14.4% glycated HbF (Figure 1A), with no haemoglobin peaks observed in the HbA or haemoglobin A_2_ (HbA2) windows. Which contrast those of Sameen et al., who showed varied HbF from 7.5% to 17.8% with normal to reduced HbA_2_ from 2.5% to 2.8% [8].

Elevated levels of HbF are typical in newborns but generally decrease to less than 2% by the first year of life [20]. If HbF levels remain high beyond this age, it may be indicative of conditions such as pregnancy, stressed bone marrow, or juvenile myelomonocytic leukaemia, though values typically do not exceed 10%. Notably high levels of HbF, combined with either absent or less than 10% HbA, are characteristic of β-thalassemia major and homozygous hereditary persistence of foetal haemoglobin (HPFH). Despite these high levels of HbF, both conditions are distinguished by their unique clinical and haematological profiles, and HbA2 levels remain normal in both disorders [21].

On the other hand, Sebia capillary electrophoresis showed a haemoglobin peak of 97.2% and a secondary peak of 2.8%, however, these peaks were not zoned, and thus both remained unlabelled (Figure 1B).

The electropherogram revealed a single dominant peak, which was not clearly separated into distinct zones and, as a result, was not labelled. This lack of zoning made it difficult to differentiate between specific haemoglobin variants. A Kleihauer-Bekte test to detect foetal cells in maternal blood was performed, demonstrating an evenly spread of HbF across all cells in the sample (pan-cellular distribution of foetal cells). This pan-cellular staining pattern effectively rule out the diagnosis of δβ thalassemia. δβ thalassemia typically presents with heterogeneous HbF distribution, often showing a patchy or uneven staining pattern across the cells [22], which was not observed in this case (Figure 1C).

Morphological analysis indicated the presence of uniform microcytic, hypochromic red cells along with target cells. No other abnormal cells were observed, and the FBC confirmed reduced MCV and MCH and this was supported by red cell morphology observed (Figure 1D).

Normal ferritin level ruled out iron deficiency and the reduced MCV and MCH suggested the possibility of co-existence of thalassaemia. The absence of HbA and HbA indicated a leaning towards a total deletion of β and δ-globin genes, along with co-existence of α(+)-thalassaemia. The phenotype of homozygotes δβ-thalassaemia with co-existing α(+)-thalassaemia dictated FBC results very similar to that of β (+) thalassaemia carrier, and Hb analysis showed >93.0% HbF with total absence of HbA and HbA_2_. The microcytosis of RBCs is caused by the co-existence of α-thalassaemia and the erythrocytosis is caused by high levels high oxygen affinity of HbF.

## Genomic Report

Gene analysis demonstrated that this patient is compound heterozygous for two large deletions in the β-globin gene cluster, consistent with the black hereditary persistence of foetal hemoglobin-1 (HPFH-1) deletion mutation and the Ghanaian hereditary persistence of foetal hemoglobin-2 (HPFH-2) deletion mutation. In addition, this patient is heterozygous for the 3.7 kb single α-globin gene deletion.

## Clinical Implications

Individuals with homozygous for δ and β deletions have reported to have erythrocytosis with a high haemoglobin level which is likely to result from high affinity of HbF. The heterozygous α(+) thalassaemia mutation may be contributing to the microcytosis and hypochromia observed in this individual.

## Reproductive Implications

These HPFH deletions do not have any reproductive implications although they cause confusion during neonatal screening if either is inherited in combination with another significant beta globin gene mutation.

The α(+) thalassaemia mutation can result in Hb H disease if inherited alongside an α(0) thalassaemia deletion and Hb H is normally associated with mild to moderate anaemia which may require clinical management in elderly patients.

## Conclusion

These findings collectively validate the presence of homozygous δβ-thalassaemia with α-thalassaemia and erythrocytosis. Through the implementation of two distinct laboratory methods, we substantiated the diagnosis, and as far as our understanding extends, this report stands as one of the limited literatures detailing this condition. Consequently, it enhances our understanding of the underlying pathogenesis of this rare disorder and its possible complications.

## Summary Sentence

This case represents a significant advance in biomedical science by highlighting a rare genetic combination that expands our understanding of thalassemia syndromes and their complex haematological presentation.

## Data Availability

The data analyzed in this study is subject to the following licenses/restrictions: The collected data cannot be disclosed owing to the confidentiality of patient information. Requests to access these datasets should be directed to sukhjinder.marwah@nhs.net.
